# Study on the Mechanism of the Micro-Charge-Detonation-Driven Flyer

**DOI:** 10.3390/mi16040441

**Published:** 2025-04-09

**Authors:** Shuang Li, Jie Ren, Chang Leng, Zhenhao Shi, Yan Ma, Mingyu Li, Qingxuan Zeng

**Affiliations:** 1 State Key Laboratory of Explosion Science and Technology, School of Mechatronical Engineering, Beijing Institute of Technology, Beijing 100081, China; ls1714513364@163.com (S.L.);; 2 China Research and Development Academy of Machinery Equipment, Beijing 100089, China; 3 Logistics Center of CALT, Beijing 100081, China

**Keywords:** copper-based azide, micro-charge, detonation-driven flyer

## Abstract

To investigate the energy transfer mechanisms during the micro-explosive initiator-driven flyer process and to guide the performance evaluation of micro-sized charges and the structural design of micro-initiators, a combined approach of numerical simulations and experimental tests was employed to study the detonation process of copper-based azide micro-charges driving a flyer. The output pressure and detonation velocity of the copper-based azide micro-charge were measured using the manganese–copper piezoresistive method and electrical probe technique, and the corresponding JWL equation of the state parameters was subsequently fitted. A simulation model for the micro-charge-driven flyer was established and validated using Photonic Doppler Velocimetry (PDV), and the influence of charge conditions, structural parameters, and other factors on the flyer velocity and morphology was investigated. The results indicate that the flyer velocity decreases as its thickness increases, whereas the specific kinetic energy of the flyer initially increases and then decreases with increasing thickness. The optimal flyer thickness was found to be in the range of 30 to 70 μm. The flyer velocity increases with the density and height of the micro-charge; however, when the micro-charge density exceeds a certain threshold, the flyer velocity decreases. The flyer velocity exhibits an exponential decline as the diameter of the acceleration chamber increases, whereas it shows a slight increase with the increase in the length of the acceleration chamber. The diameter of the acceleration chamber should not exceed the charge diameter and must be no smaller than the critical diameter required for detonation initiation of the underlying charge. The use of a multi-layer accelerating chamber structure leads to a slight reduction in flyer velocity and further increases in the transmission hole diameter while having no significant impact on the flyer velocity.

## 1. Introduction

As a key energy converter and amplifier in the micro-initiation sequence, the micro-initiator offers several advantages, including compact size, low charge mass, short detonation growth distance, high gap initiation reliability, and a simple structure. The operational process of a micro-initiator involves a multi-stage energy transfer mechanism, where the energy transfer process of the micro-charge detonation driving the flyer determines not only the output capacity of the micro-initiator but also the design parameters, such as the thickness, material, and transmission hole size, of subsequent safety mechanisms. This process is a critical factor influencing the overall parameter design and energy matching in the micro-initiation sequence. Therefore, a combined approach of experimental and simulation studies is employed to investigate the energy transfer laws of the flyer-driving process, providing both theoretical support and guidance for the performance evaluation and structural design of micro-initiators.

Numerical simulation techniques help avoid numerous hazardous explosive tests and significantly improve overall design efficiency. The accuracy of numerical simulations depends on the state equation parameters of the charge. The JWL (Jones–Wilkins–Lee) equation of state is a classical semi-empirical equation determined through experimental methods that effectively models the expansion work performed by detonation products. This approach avoids the challenges associated with performing cylindrical experiments on explosive materials [1,2]. Researchers primarily use various forms of state equations to describe the isentropic expansion of explosive products, fitting the corresponding JWL parameters. Among these, the γ-law fitting method is the most widely used approach in current research [3]. The value of the explosive adiabatic index γ determines the accuracy of the γ-law equation. Johansson [4] and Kamlet [5] have both proposed semi-empirical and semi-theoretical formulas to calculate the adiabatic index γ, but these formulas are difficult to apply to copper azide. Reference [6] obtains the adiabatic index γ of copper azide by weighting the molar components of its detonation products. In this study, the γ-law fitting method is employed to derive the JWL state equation for copper-based azide micro-charge.

The micro-initiator employs micro-charge detonation to drive a flyer, achieving energy transfer under specific gap conditions. The high-pressure pulse generated by the impact of the high-speed flyer initiates the detonation of the underlying charge. The output performance of the micro-initiator is jointly determined by the energy output of the micro-charge, as well as the material, morphology, and velocity of the flyer. References [7,8] have conducted simulations to study the effects of accelerating chamber length and charge diameter on the flyer velocity in a micro-initiator. Reference [9] used the γ-law state equation to fit the JWL state equation and studied the influence of important structural parameters on the flyer-driving process of lead azide.

In this study, by fitting the JWL state equation for copper-based azide, a combined approach of numerical simulations and experimental tests is employed to investigate the process of copper-based azide micro-charge detonation driving a flyer. The energy-matching relationship between charge parameters and flyer structural parameters is explored, providing a theoretical basis for the performance evaluation of micro-initiators and guidance for subsequent micro-initiation sequence design.

## 2. Simulation Model and Parameter Determination

### 2.1. The JWL Equation

The standard form of the JWL equation of state is given in [10]:

(1) $$P=A\left(1-\frac{\omega }{R_{1}\cdot V_{r}}\right)e^{-R_{1}\cdot V_{r}}+B\left(1-\frac{\omega }{R_{2}\cdot V_{r}}\right)e^{-R_{2}\cdot V_{r}}+\frac{\omega E}{V_{r}}$$

The isentropic equation at the C-J point is given as follows:

(2) $$P_{s}=Ae^{-R_{1}\cdot V_{r}}+Be^{-R_{2}\cdot V_{r}}+\frac{C}{V_{r}^{\omega +1}}$$

where *P* and *Ps* represent the pressure and the isentropic pressure, respectively (Pa); *Vr* is the ratio of the specific volume of the products to the initial specific volume, i.e., the relative specific volume *Vr = V*/*V_e0_*; and *E* represents the specific internal energy. The parameters *A*, *B*, *C*, *R1*, *R2*, and *ω* are the fitting parameters to be determined.

The γ-law fitting method is the most widely used approach for describing the isentropic expansion process of detonation products and fitting the corresponding JWL parameters based on Equation (1). Landau-Stanyukovich proposed a general equation of state based on a solid model of explosive detonation products:

(3) $$P=K_{A}V^{-\gamma }+f\left(V\right)T$$

where *P* represents the pressure, $K_{A}V^{-\gamma }$ represents the elastic pressure generated by molecular interactions, and *f(V)T* represents the thermal pressure due to thermal motion and vibration. When the density of the micro-charge exceeds 1 g/cm^3^, the influence of thermal pressure in the detonation products can be neglected. In this case, Equation (3) simplifies to

(4) $$P=K_{A}V^{-\gamma }=K_{A}\rho^{\gamma }$$

Equation (4) is an approximate equation of state for the detonation products of condensed-phase explosives, where *γ* is the isentropic exponent and *K_A_* and *γ* are constants related to the explosive properties [11]. By combining this with the parameter relationships on the C-J (Chapman–Jouguet) plane, the following relation is obtained:

(5) $$\left\{\begin{array}{l} \rho_{CJ}=\frac{\gamma +1}{\gamma }\rho_{e0}\\ V_{CJ}=\frac{\gamma }{\gamma +1}V_{0}\\ P_{CJ}=\frac{1}{\gamma +1}\rho_{e0}{D_{CJ}}^{2} \end{array}\right.$$

where *ρ_CJ_*, *V*_CJ_, and *P*_CJ_ represent the density, specific volume, and pressure of the detonation products at the C-J plane, *V*_0_ and *ρ*_e0_ are the initial specific volume and density of the explosive, and *D*_CJ_ is the detonation velocity. Since Equation (4) also holds at the detonation front, combining Equations (4) and (5), the following expression can be derived:

(6) $$K_{A}=P_{CJ}v_{CJ}^{\gamma }=\frac{\rho_{e0}{D_{CJ}}^{2}}{\gamma +1}{\left(\frac{\gamma }{\gamma +1}V_{0}\right)}^{\gamma }$$

Substituting the relative specific volume *Vr* into Equation (6), we obtain the *γ*-law equation of state based on the isentropic exponent *γ*, which is given by

(7) $$P=\frac{K_{A}}{{\left(V_{0}V_{r}\right)}^{\gamma }}=\frac{\gamma^{\gamma }}{{\left(\gamma +1\right)}^{\gamma +1}}\frac{\rho_{e0}{D_{CJ}}^{2}}{V_{r}^{\gamma }}$$

From Equation (7), it can be seen that once the density, detonation velocity, and *γ*-value of the explosive are obtained, the *P-V* relationship curve for the detonation products can be determined using the *γ*-law equation. The value of *γ* directly influences the accuracy of the *P-V* relationship curve and the JWL fitting results.

In this study, the *γ*-value and *P-V* curve are calculated with higher precision using experimentally determined stable detonation parameters of the micro-charge at different densities. The corresponding JWL parameters are then fitted. From Equation (5), the relationship between the *γ*-value and the micro-charge stable detonation parameters is given by

(8) $$\gamma =\frac{\rho_{e0}{D_{CJ}}^{2}}{P_{CJ}}-1$$

### 2.2. Detonation Parameter Test

The copper-based azide with different densities was prepared by controlling the reaction conditions. The detonation velocity and detonation pressure of the copper-based azide at a charge height of 1.0 mm were measured using the electrical probe method and the manganese–copper piezoresistive method. The tests were repeated three times under the same experimental conditions, and the average values and standard deviation were calculated. The results are shown in Table 1.

**Figure 1:**
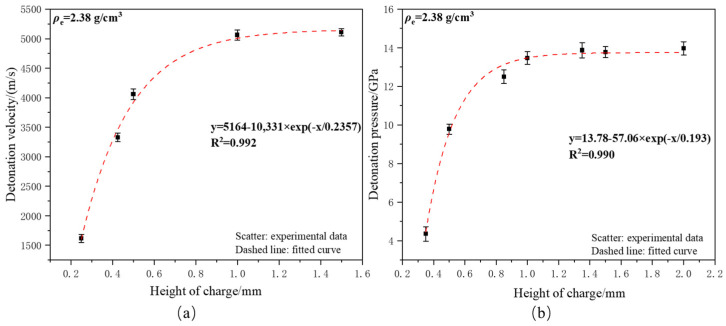
(**a**) The influence of charge height on the detonation velocity of copper-based azide; (**b**) the influence of charge height on the detonation pressure of copper-based azide.

To investigate the influence of charge height on micro-sized detonation, micro-charge detonation pressure and detonation velocity tests were conducted on copper-based azide micro-charges with the same charge density (*ρ*_e_ = 2.38 g/cm^3^) but different heights, using a firing energy of 15 V/33 μF. The tests were repeated three times under the same experimental conditions to obtain the average values and standard deviations. The detonation velocity and detonation pressure test results are shown in Figure 1.

### 2.3. JWL Parameter Determination

Based on the experimental results of stable detonation parameters for the micro-charge at different densities, the corresponding *γ*-values were calculated using Equation (8), as shown in Table 2. The results indicate that the isentropic exponent *γ* increases with density, which may be attributed to the higher proportion of metallic components in the detonation products.

**Figure 2:**
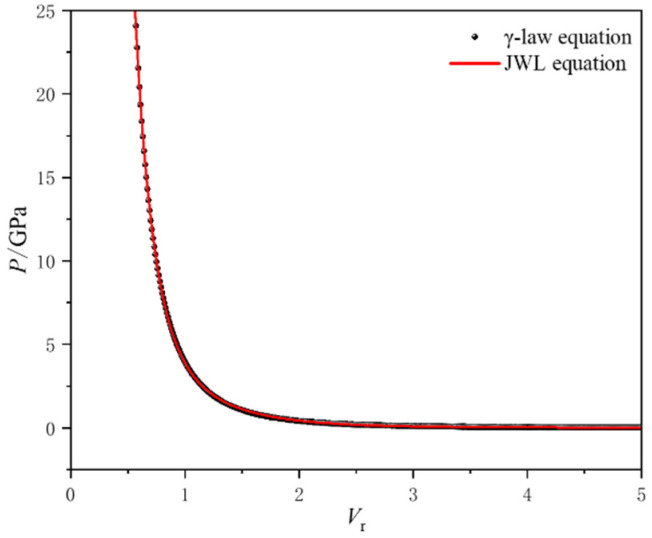
The comparison of the P-V relationship curves output by the γ-law equation and the JWL equation.

By substituting the detonation parameters and isentropic exponent *γ* values from Table 2 into Equation (7), the P-V relationship curve can be obtained. The parameters of the JWL equation are then determined through nonlinear fitting. In this study, the genetic algorithm in the 1stOpt software(Version 1.5) was used to perform parameter fitting within the high-pressure range of *V* = 1~5. A comparison of the P-V relationship curves obtained from the γ-law equation and the fitted JWL equation is shown in Figure 2. The JWL state parameters for micro-charges with different densities in a stable detonation state, based on experimental results, are listed in Table 3. The specific internal energy *E*_0_ is calculated as *E*_0_ = *ρ*_e_·*Q_v_*, where the constant-volume detonation heat *Q_v_* is given by the VPL program [12].

To study the influence of charge height on micro-sized detonation, the experimental results of the stable detonation parameters of the copper-based azide micro-charge with a density of 2.38 g/cm^3^ were used to obtain the *γ*-values and detonation parameters. These values were then substituted into Equation (7) to obtain the *P-V* relationship curve. The JWL equation parameters were subsequently determined through fitting, and the final fitted results are shown in Table 4.

### 2.4. Simulation Model

**Figure 3:**
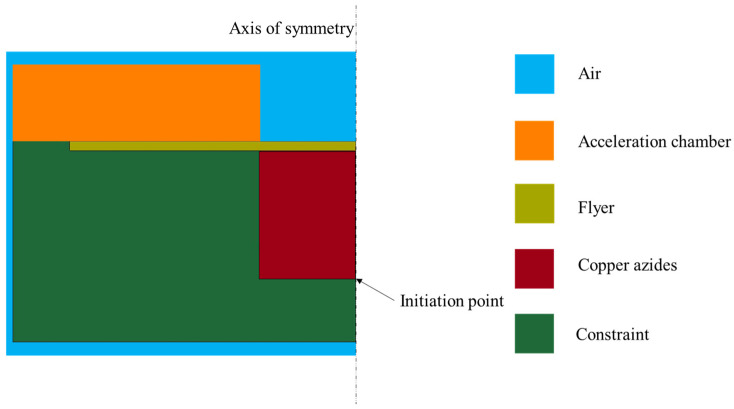
The simulation model for the copper-azide micro-charge.

The simulation model for the copper-based azide micro-charge, as shown in Figure 3, is established. Given that the device has a symmetric structure, a half-model (1/2) is adopted to simplify the simulation and improve computational efficiency. The model is constructed using actual dimensions and the μs-cm-g unit system. The grid size is set to 0.0001 mm × 0.0001 mm, with a time step of 0.001 μs and a total simulation duration of 1.5 μs. The copper-based azide is initiated by a point detonation, with the initiation point located at the center of the bottom of the charge column. The simulation model is shown in Figure 3.

Owing to the rapid expansion of detonation products during the micro-charge explosion, the Arbitrary Lagrangian–Eulerian (ALE) algorithm is utilized to define the solution domain. In this model, the copper-based azide and air domains are represented using Eulerian grids with shared nodes, whereas the flyer, constraint, and acceleration chamber structures are modeled with Lagrangian grids. The detonation process of the copper-based azide is characterized using the MAT_HIGH_EXPLOSIVE_BURN material model and the JWL equation of state, with relevant parameters listed in Table 3 and Table 4. The air domain, encompassing the entire computational space, is represented using the NULL material model and the GRUNEISEN equation of state. Within the NULL material model, the air is assigned a density of 1.2929 kg/m^3^, and the specific simulation parameters for the GRUNEISEN equation of state are summarized in Table 5.

The materials for the flyer, constraint, and base plate are titanium and polycarbonate, respectively. Both materials are modeled using the MAT_POWER_LAW_PLASTICITY material model to describe the deformation process under detonation conditions [8]. This model is based on the power-law hardening theory, which characterizes the stress–strain relationship of plastic materials such as metals. Additionally, an effective plastic failure strain (EPFS) is introduced as a threshold for element deletion. When the strain of an element grid is below the EPFS, the element remains in the calculation; when the strain exceeds the EPFS, the element is deleted, while the remaining grids continue to be computed. The parameters of the material model are summarized in Table 6.

The acceleration chamber is made of 304 stainless steel, and its behavior is described using the Johnson–Cook model and the GRUNEISEN equation of state. The material model parameters are provided in Table 7.

## 3. Experimental Verification

**Figure 4:**
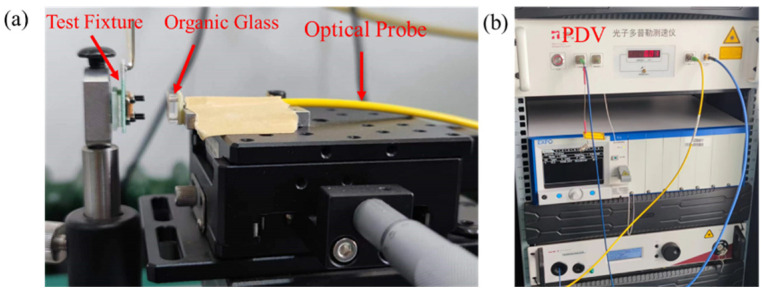
(**a**) Diagram of the experimental setup of the flyer velocity test system; (**b**) Photonic Doppler Velocimeter (PDV).

To validate the accuracy of the simulation model and material parameters, a Photonic Doppler Velocimeter (PDV) is used to capture the velocity–time curve of the flyer driven by micro-charge detonation. The flyer velocity measurement system includes a firing circuit, test fixture, optical probe, PDV, and oscilloscope. A schematic diagram of the system and its experimental setup are presented in Figure 4.

Under the influence of the firing circuit, the copper-based azide micro-charge detonates under specific loading conditions, shear-driving the flyer to accelerate and simultaneously generating a triggering signal for the oscilloscope. As the flyer accelerates, the beam splitter in the Photonic Doppler Velocimeter (PDV) divides the laser emitted by the laser source into reference light and signal light. The reference light enters the interferometer, while the signal light is directed into the high-power optical fiber isolator. The signal light, reflected from the moving object’s surface by the optical probe, undergoes a Doppler redshift in frequency. The light then passes through components such as the receiver, isolator, and interferometer, generating interference light. The photodetector measures the frequency difference between the reference light and the interference light, converting it into an electrical signal. Finally, the electrical signal is recorded by the oscilloscope.

**Figure 5:**
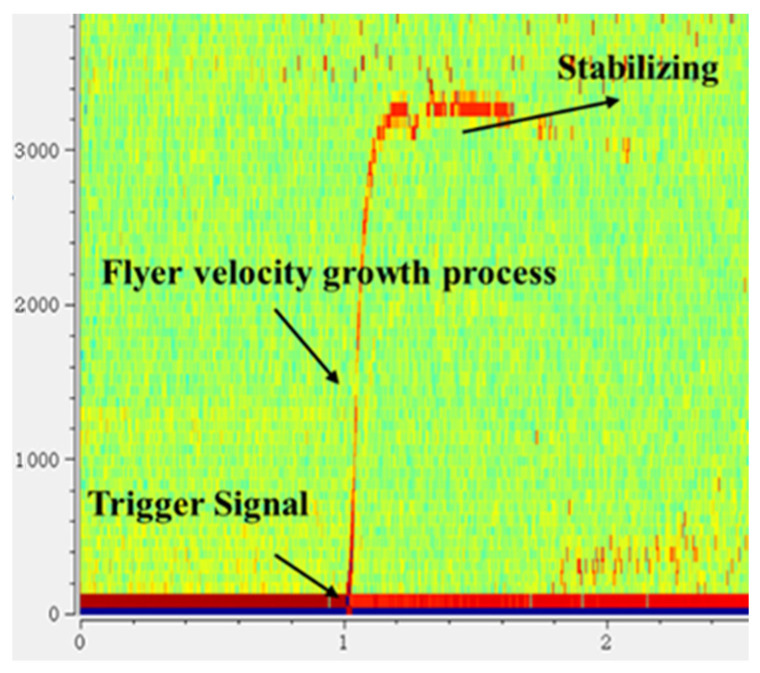
A typical flyer velocity–time curve.

To protect the PDV optical probe, an organic glass (PMMA) window is placed at the probe’s location. The raw data recorded by the oscilloscope is processed using PDVlab software (Version 1.3) to derive the velocity–time spectrum of the flyer. A typical flyer velocity–time curve is presented in Figure 5. In the figure, the intensity of the red area correlates with the strength of the signal at that point. By extracting the data points from the image, the flyer velocity curve is obtained, with a data error of ±50 m/s.

## 4. Results

### 4.1. Simulation and Experimental Results Analysis

**Figure 6:**
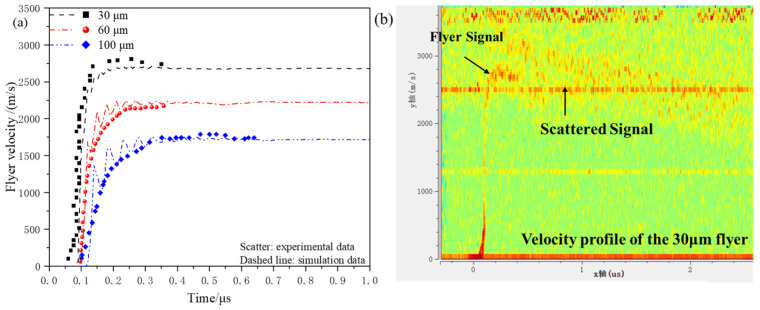
(**a**) The comparison between the experimental and simulated velocity–time curves for the flyer using Φ1.0 mm × 1.0 mm micro-charges; (**b**) PDV experimental signal.

As an example, micro-charge-driven flyers with diameters of 30 μm, 60 μm, and 100 μm were tested using a micro-charge of Φ1.0 mm × 1.0 mm. The experimental results obtained from the PDV and the simulation results under the same conditions were compared to validate the accuracy of the simulation model. The micro-charge equation of state used is the fourth JWL equation, with the diameter of the acceleration chamber being Φ1.0 mm × 0.4 mm. Figure 6 shows a comparison between the experimental and simulated velocity–time curves for the flyer. The velocity–time curves obtained from both the simulation and experiment exhibit good consistency in the overall trend, with a maximum relative error of −5.96%.

As shown in Figure 6b, the experimental curve for the 30 μm flyer differs from the typical curves of the 60 μm and 100 μm flyers. The PDV signal for the 30 μm flyer is weaker, and after reaching a steady velocity, it shows a large area of scattered signals. This could be due to significant surface deformation and changes in the laser incidence angle during the motion of the thin flyer, which degrade the PDV interference signal quality. Since the laser spot diameter from the PDV optical fiber is approximately 0.8 mm, the PDV measures the cumulative velocity signals from most of the flyer’s surface. The scattered signal indicates a significant velocity deviation at different positions of the flyer, suggesting that the flyer may have fragmented.

**Figure 7:**
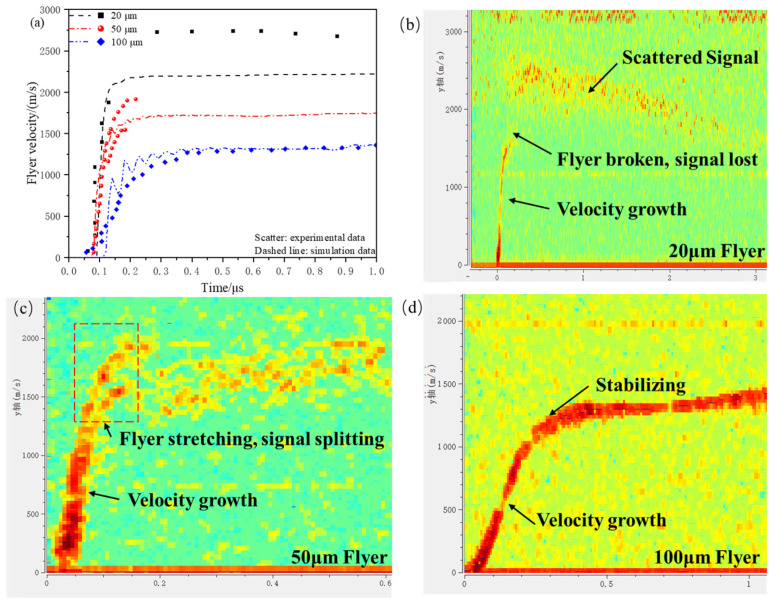
(**a**) The comparison between the experimental and simulated velocity–time curves for the flyer using Φ1.0 mm × 0.5 mm micro-charges; (**b**) 20μm Flyer PDV experimental signal; (**c**) 50μm Flyer PDV experimental signal; (**d**) 100μm Flyer PDV experimental signal.

Taking the micro-sized charge with dimensions of Φ1.0 mm × 0.5 mm driving 20 μm, 50 μm, and 100 μm flyers as an example, the simulation results are compared with the PDV experimental data under the same conditions to validate the accuracy of the simulation model. The micro-sized charge equation of state used is the one for a charge height of 0.5 mm in Table 3, with the diameter of the acceleration chamber being Φ1.0 mm × 0.4 mm. The experimental and simulated velocity–time curves for the flyer are shown in Figure 7a, while the PDV experimental data for flyers of different thicknesses are shown in Figure 7b–d. The velocity–time curves obtained from both the simulation and experiment show good consistency in the overall trend. Meanwhile, the experimental results indicate that the morphology of the flyer significantly affects the PDV test results.

Compared to the typical motion curve of a 100 μm flyer, the 20 μm flyer experiences a rapid velocity increase followed by signal loss, and then the velocity increases noticeably with the appearance of scattered signals, indicating that the flyer undergoes fragmentation. As shown in Figure 7c, as the motion distance increases, the velocity curve for the 50 μm flyer gradually splits into two, which is due to the PDV simultaneously capturing velocity signals from both the center and the edge of the flyer. This indicates that the edge stretching effect causes an increasing velocity gradient in the flyer.

The PDV experimental results show that after fragmentation, the flyer reaches a higher velocity, but with greater velocity deviation. The thickness of the flyer, charge height, and acceleration chamber collectively influence the morphology of the flyer.

### 4.2. Influence of Flyer Thickness

The flyer’s driving process is governed by both the detonation wave and detonation products, with its thickness playing a crucial role. A thinner flyer allows the detonation wave to rapidly shear it and impart higher acceleration, while the influence of detonation products remains relatively minor. In contrast, a thicker flyer exhibits lower initial acceleration, and the pressure decay due to the detonation products’ volume expansion further slows its acceleration, prolonging the time needed to reach a steady velocity.

**Figure 8:**
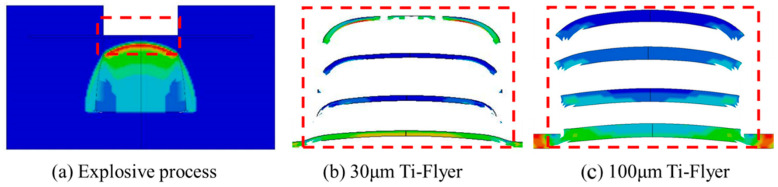
Simulation results of the shear-driving process of the flyer.

The shear-driving process of the flyer was analyzed using the results from the simulation model. The simulation results, shown in Figure 8, highlight the red and blue areas, representing high-strain and low-strain regions, respectively. A point detonation model was used to simulate the bridge film’s detonation, causing the detonation wave to propagate as a spherical wave through the copper-based azide material. Driven by the spherical detonation wave, the velocity at the flyer’s center is higher than at its edges. The shear force from the acceleration chamber causes the flyer’s edges to bend backward, while its center stretches and thins, forming an overall curved shape. Thin flyers, due to their lower mass and higher driving acceleration, exhibit a more pronounced velocity gradient. The edge-stretching effect causes fractures at the center of the thin flyer. Therefore, increasing the flyer’s thickness can enhance its integrity and flatness.

The unit area kinetic energy of the flyer reflects, to some extent, the relationship between the duration of the flyer’s action and its kinetic energy. It is defined as follows:

(9) $$e_{f}=\frac{E_{f}}{A_{f}}=\frac{m_{f}{v_{f}}^{2}}{2A_{f}}=\frac{h_{f}\rho_{f}{v_{f}}^{2}}{2}$$

where *e*_f_ represents the unit area kinetic energy of the flyer; *m_f_*, *A*_f_, and *v*_f_ are the mass, surface area, and velocity of the flyer, respectively; and *ρ*_f_ and *h*_f_ are the density and thickness of the flyer.

**Figure 9:**
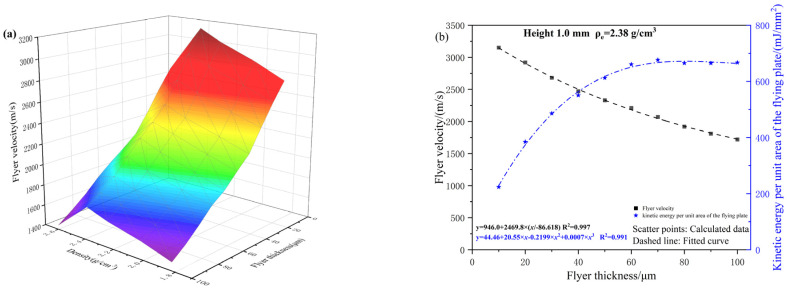
(**a**) The relationship between density, flyer thickness and velocity; (**b**) The relationship between flyer thickness, velocity and unit area kinetic energy.

To examine the effect of flyer thickness on parameters such as final velocity, the charge height and acceleration chamber conditions were held constant. The simulation model was utilized to calculate the micro-sized charge-driving process for different charge densities and to determine the steady-state velocity of the flyer at varying thicknesses. The results are presented in Figure 9a. Taking the results from the fourth JWL equation as an example, the corresponding unit area kinetic energy of the flyer was calculated using Equation (9), and the results are shown in Figure 9b.

As shown in Figure 9, when the flyer thickness is fixed, the velocity increases with increasing charge density. However, when the charge density exceeds 2.6 g/cm^3^, the flyer velocity decreases. This is because the mass and kinetic energy of the detonation products increase with charge density, thereby reducing the flyer’s velocity and kinetic energy. For a given charge density, the flyer velocity decreases with increasing thickness. This is because a thicker flyer requires more energy for shear deformation.

According to the law of conservation of energy, the total chemical energy in the micro-sized charge is converted into the kinetic energy of the flyer and detonation products, along with energy losses due to shear and expansion work on the flyer. When the total chemical energy is fixed and the parameters of the micro-sized charge remain unchanged, the flyer velocity decreases exponentially with increasing thickness. The unit area kinetic energy of the flyer initially increases and then decreases with increasing thickness. Therefore, an optimal flyer thickness in the range of 30 to 70 μm is suggested.

### 4.3. Influence of Micro-Charge Height

**Figure 10:**
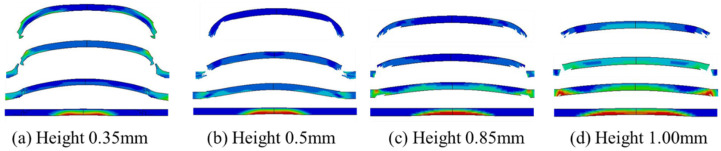
Simulation results of the shear-driving process of the flyer at different charge heights.

Since the detonation wave propagates spherically within the micro-sized charge, the charge height affects the peak pressure of the shockwave and the incident area, which, in turn, influences the final velocity and morphology of the flyer. Keeping all other parameters constant, with a 50 μm thick flyer as an example, the charge height in the simulation model was varied to analyze the corresponding shear-driving process of the flyer. The simulation results are presented in Figure 10. From the figure, it can be seen that as the charge height decreases, the area affected by the spherical detonation wave decreases, and the curvature of the flyer increases after shear formation. Thicker flyers are more likely to form a rod-shaped morphology, while thinner flyers are more prone to fragmentation.

**Figure 11:**
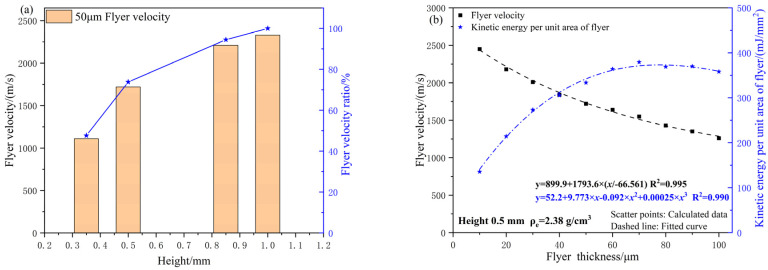
(**a**) The relationship between charge height and flyer velocity; (**b**) the relationship between flyer thicknesses and flyer velocity.

Using an acceleration chamber with dimensions of Φ1.0 mm × 0.4 mm and a 50 μm thick flyer as the research subject, the simulation model was used to calculate the effect of varying charge heights on the flyer velocity. The results are presented in Figure 11. With fixed flyer thickness, the velocity increases exponentially as the charge height increases. This trend is consistent with the detonation growth behavior observed in the experimental tests. When the charge height reaches 1 mm, the micro-sized charge achieves a stable detonation state. Further increases in charge height lead to energy losses due to radial expansion, causing the flyer velocity to approach a constant value [13].

Compared to the stable detonation state (charge height of 1 mm), the flyer velocity at charge heights of 0.5 mm and 0.85 mm reaches 73.8% and 94.5% of the maximum velocity, respectively. A comparison between charge heights of 0.5 mm and 1 mm shows that, with fixed micro-sized charge density and charge height, the relationship between flyer velocity and thickness follows a similar trend.

### 4.4. Influence of Accelerating Chamber Parameters

In the micro-charge-driven flyer process, the acceleration chamber is responsible for shearing the flyer into shape and utilizing detonation products to accelerate it. The diameter and length of the acceleration chamber directly determine the flyer’s diameter and final velocity. In the micro-detonation sequence, the safety mechanism ensures that the transfer explosion hole is misaligned with the acceleration chamber before deactivation. Once the safety is released, the transfer explosion hole aligns with the acceleration chamber. Therefore, the acceleration chamber and safety chip can be treated as a multi-layer structure.

**Figure 12:**
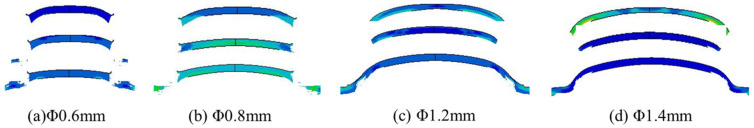
Simulation results of flyer-driven shear process with different diameters.

**Figure 13:**
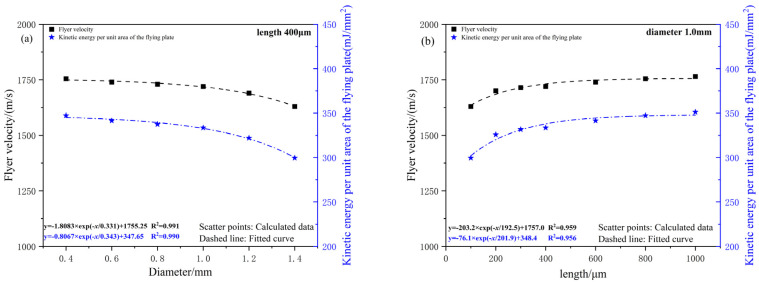
(**a**) The influence of the acceleration chamber’s diameter; (**b**) the influence of the acceleration chamber’ length.

Assuming consistent diameters for the multi-layer acceleration chambers and using a copper-based azide charge with dimensions of Φ1.0 mm × 0.5 mm and a 50 μm titanium flyer as the research subjects, a simulation model was employed to study the effects of acceleration chamber diameter and length on flyer morphology and final velocity. The influence of the acceleration chamber diameter on flyer morphology is shown in Figure 12, and the simulation results for flyer velocity are shown in Figure 13.

When the length of the acceleration chamber is fixed, the flyer’s velocity and specific kinetic energy decrease as the chamber diameter increases. This is because the chamber diameter determines the area and mass of the flyer after shear, and a lower mass is more favorable for increasing the flyer’s velocity. Driven by the spherical detonation wave, when the acceleration chamber diameter is smaller than the charge diameter (1 mm), the shear morphology of the flyer is smoother. However, when the acceleration chamber diameter exceeds the charge diameter, velocity decay becomes more pronounced. In this case, detonation products primarily act on the central region of the flyer, accelerating it while dragging the edges, which intensifies the flyer’s deformation and reduces its overall velocity. In practical applications, the relationship between the acceleration chamber diameter and charge thickness can be used to appropriately reduce the chamber diameter, ensuring the flyer’s integrity. The chamber diameter should not exceed the charge diameter, nor should it be smaller than the critical diameter for initiating the lower-level charge for the flyer.

When the acceleration chamber diameter is fixed, the flyer’s velocity and specific kinetic energy increase slightly with an increase in the acceleration chamber length. When the chamber length exceeds 200 μm, the velocity difference of the flyer does not exceed 5%. In the design of multi-layer acceleration chambers, the length of the chamber closest to the flyer should be at least 200 μm.

Considering design margins, the detonation hole diameter of the actual safety device is typically larger than the acceleration chamber diameter, resulting in inconsistent diameters in multi-layer acceleration chambers. Keeping the charge and flyer parameters constant, simulation calculations were performed to investigate the impact of variations in the diameters of the multi-layer acceleration chambers on the flyer’s velocity and other parameters. The results are shown in Table 8. The table lists the diameters and lengths of each layer in the multi-layer expansion acceleration chamber, from front to back, with the chamber closest to the flyer listed first. Simulation results indicate that the detonation hole in the multi-layer acceleration chamber slightly reduces the flyer’s velocity, while the length of the chamber closest to the flyer has a more significant impact on velocity. Further increases in the detonation hole diameter have negligible effects on the flyer’s velocity. PDV experimental results show that the velocity of an intact flyer remains relatively stable after leaving the acceleration chamber, while the velocity of a fragmented flyer decays more rapidly due to its higher speed and greater deformation.

## 5. Conclusions

This study calculates the *γ*-value and fits the *P-V* curve to determine the corresponding JWL equation parameters by measuring the stable detonation parameters of micro-charges at various densities and charge heights, all with the same charge density. A simulation model for the copper-based azide micro-charge-driven flyer was established, and its accuracy was validated using PDV test results. The maximum relative error between the simulation and experimental results was −5.96%, which confirms the reliability of both the parameter fitting and the simulation model. The following key conclusions were drawn:

(1) The velocity of the flyer decreases as its thickness increases, while the specific kinetic energy initially increases and then decreases with increasing thickness. The radial velocity gradient of the flyer is driven by the spherical detonation wave, and when the flyer is excessively thin, it is prone to fragmentation. An optimal thickness range of 30–70 μm for the flyer is therefore recommended.
(2) When the thickness of the flyer is fixed, the velocity of the flyer increases with both the micro-charge density and the charge height. However, if the micro-charge density becomes excessively high, the flyer velocity decreases. The charge height influences the peak shock pressure and the incident area, which, in turn, affects both the flyer velocity and its morphology. A relationship exists between the charge height and the diameter of the acceleration chamber. The diameter of the acceleration chamber should be appropriately reduced in accordance with the charge height to ensure the integrity of the flyer.
(3) The flyer velocity decreases exponentially as the diameter of the acceleration chamber increases, while it increases slightly with an increase in the length of the acceleration chamber. The diameter of the acceleration chamber should not exceed the charge diameter and must not be smaller than the critical diameter required to initiate the subsequent charge of the flyer. The multi-layer acceleration chamber structure leads to a slight reduction in the flyer velocity, and further increases in the detonation hole diameter have negligible effects on the flyer velocity.

## Figures and Tables

**Table 1:** The test results of the detonation velocity and detonation pressure of copper-based azide at a charge height of 1.0 mm.

Number	Density (g/cm^3^)	Detonation Pressure/GPa	Standard Deviation	Detonation Velocity/(m/s)	Standard Deviation
1	1.82	9.62	0.33	4673	57
2	2.01	11.63	0.40	4982	45
3	2.24	13.15	0.41	5054	56
4	2.38	13.78	0.35	5164	62
5	2.60	12.24	0.29	4714	46

**Table 2:** The isentropic exponent (γ) of copper-based azide micro-charge at different densities.

Number	Density (g/cm^3^)	Detonation Pressure/GPa	Detonation Velocity/(km/s)	γ
1	1.82	9.42	4.67	3.22
2	2.01	11.63	4.98	3.29
3	2.24	13.15	5.05	3.35
4	2.38	13.78	5.16	3.61
5	2.60	12.29	4.71	3.69

**Table 3:** The JWL state parameters of copper-based azide micro-charge at different densities.

Number	A/GPa	B/GPa	R1	R2	ω	E0 (10^5^ J/cm^3^)
1	702.5	16.04	6.219	1.753	0.2001	0.0584
2	662.5	17.38	5.730	1.736	0.2000	0.0643
3	860.5	17.27	5.623	1.724	0.2001	0.0725
4	748.2	18.43	5.476	1.758	0.3172	0.0777
5	678.7	8.90	5.314	1.547	0.2159	0.0681

**Table 4:** The JWL state parameters of copper azide micro-charge at different charge heights.

Height/mm	A/GPa	B/GPa	R1	R2	ω	E0 (10^5^ J/cm^3^)
0.35	320.3	7.00	5.999	1.628	0.4999	0.0223
0.5	398.2	15.03	5.707	1.598	0.2243	0.0594
0.85	683.1	20.86	5.577	1.763	0.3893	0.0639
1.0	748.2	18.43	5.476	1.758	0.3172	0.0777

**Table 5:** The simulation parameters for the GRUNEISEN equation of state.

EOS_GRUNEISEN (cm-g-μs)							
Material	C	S1	S2	S3	GAMAO	A	E0
air	0.4569	1.49	0	0	2.17	0.46	0

**Table 6:** Material model parameters for the titanium and polycarbonate.

Material	ρ/g·cm^−3^	E/GPa	G/GPa	PR	k/GPa	*n*	EPFS
titanium	4.51	113.76	43.76	0.30	0.909	0.123	0.717
polycarbonate	1.19	2.34	0.85	0.38	0.115	0.192	3.016

**Table 7:** Material model parameters for the acceleration chamber.

**304 stainless steel**	**MAT_JOHNSON-COOK**	**A**	**B**	***n***	**c**	**m**	**CP**	**D1**
		0.792	0.510	0.26	0.014	1.03	0.477	0.8
	**EOS_GRUNEISEN**	**C**	**S1**	**S2**	**S3**	**GAMAO**	**A**	**E0**
		0.4569	1.49	0	0	2.17	0.46	0

**Table 8:** The effect of a change in the diameter of a multi-layer acceleration chamber on the velocity and kinetic energy per unit area of the flyer.

Diameter/mm	Length/μm	Velocity/(m/s)	Kinetic Energy per Unit Area/(mJ/mm^2^)
1.0 + 1.4	200 + 400	1690	322.0
1.0 + 1.4	400 + 200	1710	329.7
1.0 + 1.8	200 + 400	1690	322.0
1.0 + 1.8	400 + 200	1710	329.7
1.0 + 2.2	200 + 400	1690	322.0
1.0 + 2.2	400 + 200	1710	329.7

## Data Availability

The original contributions presented in this study are included in the article. Further inquiries can be directed to the corresponding author.
